# The sustainability effects of two reading interventions on Saudi nursing students’ comprehension of scientific research

**DOI:** 10.1371/journal.pone.0309898

**Published:** 2024-10-24

**Authors:** Modi Al-Moteri, Ibrahim R. Alqarni, Ahmed AbdElbagy Ibrahim Elryah, Virginia Plummer, Mohammed Almalki

**Affiliations:** 1 Medical Surgical Nursing Department, College of Nursing, Taif University, At Taif, Saudi Arabia; 2 Department of English Language and Translation, College of Languages and Translation, King Saud University, Riyadh, Saudi Arabia; 3 Institute Health and Well-Being, Victoria, Australia; 4 Federation University, Berwick, Australia; Management and Science University, MALAYSIA

## Abstract

**Background:**

Scientific literature is presented in complex language, most frequently in English, and includes technical jargon that represents a challenge to comprehension of an English as a foreign language (EFL) nursing student. Yet scientific literature is a powerful and trustworthy source of evidence to guide nursing practice.

**Purpose:**

The aim is to examine two reading interventions (Translation vs Synthesization) and to determine which one produces long-term sustainability effects in scientific research reading comprehension.

**Method:**

A two-group posttest-only randomized comparative design was used in which 120 participants were randomly assigned to two groups. Several instruments were used to collect the data.

**Results:**

Study findings showed that the synthesization group significantly produced better results when compared with the translation group on both the immediate (*p* = 0.01) and the delayed (*p* = 0.013) reading comprehension tests. It shows also that gender differences have a significant impact on reading comprehension with a favor to males in the long-term reading comprehension outcome (*p* = 0.038) of synthesization and females in the short-term reading comprehension outcome (*p* = 0.015) of translation. English proficiency was significant with determination, metacognitive, and social skills in the synthesization group (p = 0.00, p = 0.01, p = 0.007 respectively).

**Conclusion:**

The results suggested that synthesization could be an effective reading approach in improving EFL nursing students’ reading comprehension of scientific literature.

## Introduction

With the growth of the importance of scientific research literature in improving the quality of practice, [1, 2] the need to prepare nursing students proficient in use the research evidence, has risen dramatically [3]. Nurses are expected to understand and apply scientific research literature to clinical practice [4]. The ability to utilize scientific research literature not only improves the quality of care and patient outcomes but also assists professional growth for nurses [5]. However, scientific research literature is presented in complex scientific language, generally in English, and includes technical terminology that may impair second language speakers’ ability to comprehend and appropriately process scientific information [6]. Indeed, scientific terminology was noted as a critical problem in the lack of understanding of scientific knowledge [7, 8]. Nurses need not only to be competent in general English but also competent in scientific terminology, which is nearly a second language per se [7].

Most Bachelor of Science degrees require students to read research literature, which is not commonly essential part of their study plan [9]. Scientific research in nursing programs is usually introduced through the program courses in the form of findings, with a high expectation that students will be competent to deal with the research literature at graduation [10]. For example, according to the recent requirements, registered nurses at the time of employment should be prepared to use and apply scientific research literature [11]. Studies have shown that nursing students do not have the required skills to use scientific research [12].

In the context of English as a foreign language (EFL), students in general need reading comprehension strategies since English is universally utilized and is a key factor in the students’ successful professional development [13]. It is worth noting here that there is international call for adopting new reading strategies to upskill nursing students in the area of scientific research literature [14]. Within most nursing programs in Saudi Arabia, translation-based reading strategy continues to be the most popular tool used in English reading comprehension [9]. Translation is defined as the process of “producing one language based on the knowledge of another language” [15]. However, translation has always been an area of controversy between supporters and opponents [16].

Zimmermann and Hutchins [17] suggest that reading is when readers are involved with the text and “really think about what they are reading”. Reading comprehension strategy is the process of utilizing different skills such as “selecting key information”, “organizing, and summarizing ideas and information”, and “correcting comprehension breakdowns” [13]. These skills suggest that comprehension is mainly a consequence of certain cognition and metacognition processes and that EFL students use a vast number of processes as they interact with the scientific research literature. These cognitive and related metacognitive processes demonstrate a strategic approach to build a coherent understanding of the scientific research literature.

Synthesization reading strategy is believed to include cognition and metacognition skills [13]. Synthesization is defined as the process of “pulling together background knowledge, newly learned ideas, connections, inferences and summaries into a complete and original understanding of the text" [18], which make it appropriate for reading comprehension of scientific research literature. The synthesization strategy was proposed in 2003 by Zimmermann and Hutchins. Recently, Zimmermann and colleagues re-reviewed the reading comprehension strategies and assert that the strategies are still effective [19].

Recently, reading comprehension strategies have been given great attention [20]. The challenges facing the EFL nursing students in reading and understanding scientific research are ongoing major concerns for the nursing educators. Despite the fact that all non-native English speaking nursing students receive comprehensive English courses prior to their enrollment in the nursing program, students appear to lack strategies necessary to achieve better comprehension [21]. However, most of reading studies investigate comprehension in the first language (L1), and few examine reading comprehension in EFL [22]. Reading research also tends to assess the immediate outcome of reading comprehension following the intervention to evaluate the intervention effects, and few collect follow-up data to assess the long-term impact of the intervention [23]. Moreover, less is known about reading comprehension strategies that are appropriate for EFL nursing students to enable them to construct meaning from scientific research literature [2, 24].

This study selected two reading comprehension strategies: Translation reading strategy (mostly used by EFL) and Synthesization reading strategy. Scientific texts pose particular mental challenges and the aim was to examine which of these two strategies produces long-term sustainability effects in scientific research reading comprehension. Long-term effect is defined in this study as the data collected one week after the end of the reading interventions.

This study draws on the theoretical framework of successful comprehension proposed by Research and Development [25] as a basis for guidance in study questions, methods and analysis.

### Theoretical framework

The Reading Study Group (RSG) has framed successful reading comprehension around the interaction of three dimensions: the reader, the text, and the activity of reading [19]. The three interrelated dimensions (reader, text, activity) occur within a larger context that forms and is reformed by the reader and interacts with each of the dimensions iteratively throughout the process of reading [21]. Hence, reading comprehension success requires examining data in relation to what ‘reader’ can bring to the act of reading (cognitive abilities, social skills, memory etc.); the features of the text being read (complexity, familiarity, wording, representation); the reading activity (actions to process the text, outcomes of the reading); and the socio-cultural context surrounding the reading activities [25].

### Reader

The first component in the process of reading comprehension is focused on the reader. Indeed, the reader brings to the act of reading comprehension a number of cognitive, social and academic capabilities such as (1) determination, (2) memory, (3) cognition, (4) metacognition and, (5) social abilities [26]. These abilities considerably vary among EFL nursing students and even within an individual reader. In 2022, Baixinho [11], et al. claimed that nursing students were not receiving much educational instruction on how to comprehend scientific research literature. This claim is well supported as the literature continuously showed a lack of scientific research literature implementation at a desirable level [27, 28]. This may mandate making efforts to identify cognitive skills that can be taught to nursing students to increase their understanding and memory for scientific research literature [29]. Recent studies have revealed limited chances for nursing students’ experience reading pedagogical strategies of scientific research literature with the most often cited barriers were lack of understanding of statistics and interpretation of results [30, 31]. Many nursing educators do not consider the development of pedagogical strategies for scientific research literature as an important part of nursing education [32]. Consequently, a paucity of research focused on developing educational programs to promote scientific research literature in the nursing literature was found [33, 34].

### Text

The second component in the process of reading comprehension focuses on the text. EFL nursing students encounter comprehension difficulties when reading scientific English text that is different from a general English text [4]. Scientific vocabulary, which has an important role in scientific research, consists mainly of words that occur across medical fields, and differ from language found in general English texts [35]. The most typical features of specialized nursing scientific English text are the nursing and medical terminology, long sentences, use of passives and grammatical skills that fit with hypotheses formation and probabilistic terms expression [36]. Unfortunately, although reading comprehension of scientific English text in higher education has been the focus of investigation for decades, [37, 38] the specific reading comprehension experiences, requirements and challenges of nursing students have remained largely overlooked [38].

Clinton et al. [39] described a myth related to the lack of proficiency in reading comprehension of scientific English text. The myth asserts that reading comprehension is a function of text factors. Indeed, making straightforward inferences from a general English text is easier than incorporating ideas, interpreting and evaluating textual elements of scientific English text [39]. Yang [35] claimed that without access to discipline-specific scientific word lists, it is highly unlikely that EFL nursing students’ reading and scientific writing proficiency will be enhanced. However, the Yang [35] study gave a focused attention to the topic of EFL nursing students in relation to nursing discipline scientific texts, and no scientific research literature reading comprehension was reported. Text must be supplemented by appropriate activities, so called, ‘instructional strategy’ to support the reading purpose, which bring us to the last component in the process of reading comprehension, the activity.

### Activity

One of the primary activities for helping nursing students learn is reading activity itself. The reading activity is a “mental tool” that help readers to interact with a specific written text to comprehend the meaning [40]. It is a task function (i.e. the instructions that guide the reader’s engagement with text) used to enhance comprehension during reading [41]. Gonçalves et al. [42] asserted that depending on the type of text, reading activities can vary, ranging from scanning to inferencing, also added that the outcomes of reading are part of the reading activity itself and may include short and long-term reading comprehension outcomes. Many reading activities have been applied by researchers to promote general English comprehension, with good results [41].

### Context

The socio-cultural factors (context) where the interaction of the three essential components take place, surround the interactive processes and act as an important variable at the core of reading comprehension [43]. The context may extend to the reader (background, gender, age), linguistic experience (proficiency), and discipline (science, literature).

### Study aim and questions

The dominant focus of the current study is to examine “translation and synthesization” reading strategies and to determine their long-term impact on scientific research literature reading comprehension. Several unresolved queries that relate to the three essential components of reading comprehension (reader, text, context), will be explored in relation to “translation and synthesization”. It worth noting here that text and activity will be treated as one unit of analysis, for the investigation of reading comprehension outcomes. Researchers who support this point of view assert that text cannot be isolated from the activity in which reading comprehension of the text is taking place [43]. Text and reading activity can be easily manipulated—in comparison to reader (e.g., abilities, interest) and contextual factors (e.g., gender, age, experiences), which are generally more explainable in specific learning environments [43]. It is therefore, the interaction of the demands—the task-relevant features of the text—that makes one text activity more or less difficult than another [44].

The followings are the research questions:

### Context

1. Is there any statistically significant difference between readers’ demographic data and experience and the short-term and long-term scientific research comprehension in relation to the intervention received?

### Reader

2. Do cognitive, determination, social, and academic capabilities interact with readers’ background and experience in the two intervention groups regardless to the comprehension outcomes in such a way to produce a statistically significant effect?

### Text- Activity

3. Which reading intervention creates a long-term effect on students’ comprehension of scientific research?

## Methods

A two-group posttest-only randomized experiment design was used. In this context, the current study utilized immediate and delayed posttest assessment of reading comprehension with two sample groups and because the study used random assignment, it can be assumed that the two groups are “probabilistically equivalent” to start with and without the pretest [45]. Considering the study aim, which focuses on examining the effect of two different reading strategies on scientific research literature reading comprehension, this design is appropriate. Indeed, the two-group posttest-only randomized experiment design allows examination of whether two different text-activities would lead to a statistically significant change on the related group at the end of the interventions and which group is better in term of comprehension test score.

### Study setting and recruitment

The study was conducted at a public Nursing College in Saudi Arabia in December 12^th^, 2021 to December, 26^th^ 2021. Participants were considered eligible if they were enrolled in the Bachelor of Nursing program, and were involved in clinical practice. All third- and fourth-year nursing students (N = 253) were initially considered eligible. A convenience sampling technique that helps to identify those who are interested in participation in the study, was used at first. Of the 253 nursing students, 120, (Male = 60 and Female = 60) agreed to participate in the study by signing a written consent. Participants age ranged from 20 to 23 years old. They were informed that their participation will contribute to the development of the teaching techniques. They were also instructed that the study results would have no bearing on their formal evaluation. Potential participants were also informed that (i) the study will include no personal information, (ii) they are free to participate and (iii) that signing their names is considered consenting on participation, however, they are free to withdraw at any point by sending a withdraw email request to the primary researcher.

Random sampling was then used to randomly divide participants into two groups: (1) Translation group (n = 60 participants); and (2) synthesization group (n = 60 participants) (see Table 1). The age of synthesization group ranged from 20 to 23 with Mean = 21.2 year and SD = 0.66 and for group translation, participants age ranged from 20 to 25 with Mean = 21.2 year and SD = 0.89.

**Table 1:** Sample description.

Population	Total number of participants	Gender	Native language	Major	Academic year	Groups
253	120	Female (n = 60) Male (n = 60)	Arabic	Nursing	2021–2022	Translation (n = 60) Synthesization (n = 60)

To maintain follow up during the study steps and to further ensure anonymity, researchers, held a meeting session with these participants (n = 120) to clarify any concerns.

To investigate the impact of participants’ characteristics and background on their reading comprehension of scientific research, a descriptive analysis was undertaken.

### Data collection tools

A number of instruments were employed to assess EFL nursing students’ reading comprehension.

### Context

Reader characteristics and background survey consisted of six items to provide a richer context for understanding the data. The survey sought information about participants’ characteristics and background including their gender, age, if they had additional English courses in the previous year; and their self-reported overall English proficiency. The background data also included information about participants clinical experience. Further, participants were asked if they were familiar with scientific research literature.

### Reader

Schmitt’s vocabulary learning strategy (VLS) taxonomy questionnaire was used to assess readers’ cognitive capabilities that may impact EFL nursing students’ reading comprehension of scientific research literature. The survey was adapted from Schmitt’s vocabulary learning strategy taxonomy [46] to investigate English learning strategies. The VLS is a self-report questionnaire that consists of five domains with 40 items in which participants rate their vocabulary learning strategy on a Likert scale using a 5-point scale ("never," to "always"). The VLS contained questions about the determination strategy (8 items), cognitive strategy (8 items), memory strategy (8 items), social strategy (8 items), and metacognitive strategy (8 items). As for the reliability coefficients, the VLS was .924, ranging from .590 to .853 for the 5 domains. The academic capabilities of the participants were assessed through their Grade Point Average (GPA).

### Text

The Jocelyn Chew et al. [47] scoping review was employed for reading strategies in the current study—and was suggested by the Medical-Surgical academic instructors, as a learning activity for promoting evidence-based practice literacy. This scoping review concerned “to identify current research on turning frequencies of adult bed-bound patients and inform future turning practices”. The topic of this scientific research is important for nursing practice, however the number of times of repositioning to prevent hospital-acquired pressure ulcers is an area of extensive debate. The review updates the current practice of repositioning and brought new evidence to nursing literature. Nursing students in the current study were clearly informed that the purpose of the reading task was to identify the best practices to prevent hospital-acquired pressure ulcers. The abstract, aim, discussion and conclusion sections of the selected scientific research, were all hidden purposively, so students only had a background knowledge from the literature and data analysis sections only. This requires the student to have a reasonable level of syntax, vocabulary and background knowledge.

### Reading strategies

Zimmerman and Hutchins’ [17] instructions for reading comprehension was given to the participants of the synthesization group, to guide them through the synthesizing reading activity. Meanwhile, the unified medical dictionary, English-Arabic [48] was given to the participants of the translation group, to guide them through the translation reading activity.

### Reading comprehension assessment tools

Comprehension tests were used to assess the impact of each of text-activity—translation and synthesization interventions on EFL nursing students’ reading comprehension of scientific research. Two types of comprehension tests were used—short and long-term comprehension impact tests.

### Short-term reading comprehension

To investigate the short-term outcomes of the intervention on the comprehension of the scientific research, five items, two true/false questions (T/F-Q) and three multiple-choice questions (MCQ), were constructed. The items were written with a focus on the scientific research outcomes. Thus, the constructed items aimed to evaluate the ability of participants to identify and understand the key findings of the scientific research. The comprehension test score was calculated by comparing participant’s answers with answer sheet prepared by researchers for each item (correct or incorrect).

### Long-term (1 week) reading comprehension

A one case vignette was conducted to evaluate the long-term (1 week) comprehension effects of the different type of text-activities on using the scientific evidence in clinical decision making. Thus, prior to constructing the case vignette, the concept of utilization of the scientific evidence in the light of nursing practice was defined as the ability of the students to use the scientific evidence to guide clinical decision making when deciding upon a nursing action.

### The reliability of the two comprehension tests

Prior to conducting data collection, the opinion of three subject matter experts (SMEs), were sought. SMEs were full time lecturers of ’Adult Nursing Care’ with an over 10-year teaching experience. SMEs were instructed to review the clarity of the items first, and then score the reliability of each item for the two comprehension tests (short-term and long-term). Inter-rater reliability was important to identify the degree of agreement among SMEs concerning the test items. Consistency estimates of the inter-rater reliability technique was selected in this study to measure inter-rater reliability. Based on Gisev et al [49] the result of interrater reliability (0.81) was considered almost perfect.

### The procedure

Researchers wished to meet the appropriate ethical standards for the current study by maintaining anonymity and confidentiality. However, because the current study consisted of two phases with short (hours) and long (one week) breaks, participants were informed that each participant response is connected to specific individual research code e.g., Gr1-P1, Gr1-P2…Gr2-P1, Gr2-P2 and so on, and that students must ensure using the same research code when they access the survey link in the second phase. Participates were informed that these research codes do not give researchers any clue about the participants’ identities, but allow them to follow up with participants responses throughout the research phases.

Step 1: For the two groups, the procedure included several steps. Prior to conducting the intervention, participants were divided into translation and synthesizing groups, provided the codes, and instructed to fill participants’ characteristics and background survey and Schmitt’s vocabulary learning strategy (VLS) taxonomy questionnaire.

Step 2: In two separate classrooms with two instructors present in each class at all times, each participant in each group was then given the scientific research (see Data collection tool section, Text part). Participants were given two hours to complete reading the scientific research.

Step 3: The two groups received different instructions according to the reading strategy type; however, they were kept unaware of the actual aim of the study so that their awareness would not affect the results of the experiment adversely. The translation group was instructed first to individually read the scientific research and highlighted all the unfamiliar words and terms and translate them into Arabic language before starting to work on reading comprehension. The synthesization group, however, were offered no Arabic translation. The synthesization group was encouraged to slowly read the scientific research and ask themselves questions such as, "What the scientific research is about?". They were encouraged also to underline any of the difficult and unfamiliar technical jargons and try to make a logical guess of the meaning based on the textual clues. They were instructed to connect the pieces of information that they gathered from different parts of the research evidence with their previous knowledge in order to understand the meaning.

Step 4: A link including the first comprehension test was administered immediately after the completion of the reading task. A five minutes time period was determined to be appropriate for participants to answer the items of the short-term impact comprehension test. A week later all nursing students who participated in the study were grouped in a classroom and a barcode with a URL embedded, showed to them. They were instructed to scan the barcode, add their individual research code and answer the test item. A one-minute time period was given to the participants to answer the item of the long-term impact (1week) comprehension reading test.

### Ethical considerations

Ethical approval was granted by the Ethics Review Committee (ERC) of the college. Participants were informed that their participation was entirely voluntary and that any information resulting from the study was treated with confidentiality.

## Data analysis

Data analyses were administered using SPSS 23.0. The demographic questionnaire was analyzed by an analysis of frequency. Percent of categorical variables were compared using Chi-square test or Fisher Exact test when appropriate. Pearson’ correlation coefficient was calculated to assess relationships between various study variables, with (+) sign to indicate direct correlation and a (-) sign to indicate inverse correlation; values near to 1 indicated strong correlation and values near 0 indicated weak correlation. All tests were two sided with p-value < 0.05 considered statistically significant, p-value < 0.001 was considered statistically highly significant, p-value ≥ 0.05 was considered statistically insignificant.

## Results

GPA of group synthesization ranged between 1.8 and 3.92 with Mean = 3.2 and SD 0.47 and for group translation ranged between 1.23 and 3.89 with Mean = 3.1 and SD = 0.59. This mean that both groups were almost at the same level of academic performance as reflected by the GPA.

Chi-square test and Fisher Exact test were then used when appropriate to answer the first research question and identify whether a statistically significant association existed between participants’ demographic data and comprehension outcomes (short-term and long-term) in relation to translation and synthesization interventions. Table 2 shows the statistically significant findings between gender and synthesization and translation for long-term (p = 0.038) and short-term (p = 0.015) reading comprehension outcomes respectively.

**Table 2:** Relation between short-term and long-term reading comprehension outcome of text-activities and demographic characteristics.

Items	Synthesization				Translation			
	reading comprehension				reading comprehension			
	Short-term		Long-term		Short-term		Long-term	
	χ ^2^	p-value	χ ^2^	p-value	χ ^2^	p-value	χ ^2^	p-value
Age	f	0.12	f	0.99	f	0.23	f	0.99
Gender	0.62	0.43	4.3	0.038	5.9	0.015	0.8	0.37
English courses	f	0.29	f	0.74	f	0.99	f	0.67
Clinical experience	0.27	0.61	0.23	0.63	0.33	0.57	3.6	0.058
Research experience	f	0.69	f	0.4	2.3	0.32	0.44	0.8
Overall English proficiency	0.04	0.68	0.32	0.85	0.42	0.81	0.22	0.89

χ ^2^:Chisquare test f:Fisher exact test p>0.5:no significant p<0.5:significant

The second research question focused on assessing whether a statistically significant association existed between the participants’ cognitive, social and academic performance capabilities and demographic data in the two groups regardless to the comprehension outcomes; this was investigated using the Chi-square test and Fisher Exact test and displayed in Table 3. The results revealed that clinical experience was statistically significant with determination strategy in the synthesization group (p = 0.03). Additionally, the English proficiency effect was significant with determination, metacognitive and social skills in synthesization group (p = 0.00, p = 0.01, p = 0.007), indicating the interactive role of reading strategies factor, key reader characteristics and capabilities, in the scientific research comprehension. Meanwhile, gender (p = 0.035) and English proficiency (p = 0.046) were both statistically significant with metacognitive skills in the synthesization group.

**Table 3:** Relation between participants’ characteristics and their cognitive and social abilities in relation to the two different interventions.

	Items	Intervention			
		Synthesization n.60		Translation n.60	
		χ ^2^	p-value	χ ^2^	p-value
Determination skills	Age	f	0.42	f	0.99
	Gender	0.07	0.79	1.9	0.16
	English courses	f	0.73	f	0.99
	Clinical experience	4.5	0.034*	0.039	0.84
	Research experience	f	0.17	2.2	0.83
	Overall English proficiency	14.3	0.001*	0.14	0.93
Cognitive skills	Age	2.6	0.28	f	0.99
	Gender	2.3	0.32	3.7	0.053
	English courses	3.6	0.14	f	0.99
	Clinical experience	1.4	0.51	f	0.302
	Research experience	4.6	0.103	1.1	0.59
	Overall English proficiency	2.4	0.66	1.7	0.43
Memory skills	Age	0.58	0.75	f	0.66
	Gender	2.3	0.32	1.4	0.24
	English courses	1.4	0.49	f	0.67
	Clinical experience	1.3	0.51	0.17	0.68
	Research experience	0.25	0.88	3.8	0.15
	Overall English proficiency	6.8	0.15	3.3	0.19
Metacognitive skills	Age	1.3	0.53	f	0.99
	Gender	3.4	0.18	4.4	0.035*
	English courses	0.77	0.68	f	0.25
	Clinical experience	3.9	0.14	1.3	0.26
	Research experience	3.4	0.18	0.75	0.69
	Overall English proficiency	18.4	0.001*	6.1	0.046*
Social skills	Age	0.48	0.79	f	0.99
	Gender	0.32	0.85	1.3	0.26
	English courses	0.88	0.64	f	0.33
	Clinical experience	1.3	0.53	0.35	0.55
	Research experience	1.4	0.5	3.6	0.16
	Overall English proficiency	13.9	0.007*	1.9	0.39

χ ^2^:Chisquare test f:Fisher exact test p>0.5:no significant

*p<0.5:significant

Whether a statistically significant association exists between participants’ academic performance and their cognitive and social abilities in the two interventions, regardless of the comprehension outcomes, was investigated using Pearson’ correlation coefficient. As revealed by Table 4, the only statistically significant relationship was with the metacognitive abilities (p = 0.0001) in synthesization.

**Table 4:** Relation between participants’ cognitive and social abilities and academic performance in relation to the two different interventions.

Items	Academic performance			
	Synthesization		Translation	
	r	P	r	p
Academic performance	1		1	
Determination skills	0.231	0.076	0.152	0.245
Cognitive skills	0.168	0.2	0.037	0.777
Memory skills	0.064	0.629	-.003	0.981
Metacognitive skills	.447**	0.0001	0.133	0.31
Social skills	0.039	0.766	0.065	0.621

(r) correlation coefficient

** Correlation is significant at the 0.01 level

* Correlation is significant at the 0.05 level.

The third research question intended to identify which of the two interventions produced long-term impact on the scientific research reading comprehension, in order to determine which reading comprehension strategy works best with EFL nursing students. This was analysed by calculating the relative change (RR) between the studied groups by identifying the ratio of the probability of an outcome in translation group to the probability of an outcome in synthesization group (Table 5). The short-term and long-term impact of comprehension was 1.67 and 1.87 time \respectively in synthesization group compared to the translation group (p<0.01 and p<0.013).

**Table 5:** Short-term and long-term reading comprehension tests results.

	Studied groups				χ ^2^	p-value	RR (95%CI)
	Synthesization (n = 60)		Translation (n = 60)				
	No.	%	No.	%			
Short-term outcome							
accepted> = 60%	35	58.3	21	35.0	6.6	0.01(S)	
Unaccepted <60%	25	41.7	39	65.0			1.67(1.1–2.3)
mean ±SD range	2.6±1 1–5		2.3±0.92 0–5				
Long-term outcome							
Correct	28	46.7	15	25.0	6.1	0.013(S)	1.87(1.1–2.2)
Un correct	32	53.3	45	75.0			

χ 2 chi square test of significant (S):p<0.05 significant RR: relative improvement (CI):confidence interval

## Discussion

Reading comprehension for EFL is a complex process that consists of separate components that relate to each other to create a comprehension outcome [44]. Reading comprehension of scientific research literature may challenge undergraduate nursing students [38]. Consequently, EFL nursing students tend to avoid the utilization of these sources of information in clinical practice [27]. The nursing literature has included reports focused on examining nurses’ scientific research literature literacy [50], perception [11] and the searching process [3], but few studies focused on studying EFL nursing students’ reading comprehension of scientific research literature [33, 34].

The current study aimed to examine two reading comprehension strategies—Translation and Synthesization—and to see which one produces long-term sustainability effects in scientific research reading comprehension. In general, the study result showed that EFL nursing students who went through synthesization intervention had a relative improvement of short-term (1.67) and long-term (1.87) reading comprehension outcomes in contrast to the translation intervention; these results attained statistical significance, suggesting that the synthesization intervention effect was sustained at the follow-up time point. In other words, the study results indicate that EFL nursing students in the synthesization intervention maintained their reading-related gains over time and thus this reading intervention maybe the appropriate to enable students to construct meaning from scientific research literature. Synthesization allows students to analyse, gauge and reflect on what they are reading [51]. Meanwhile, translation has been an area of debate among linguistics experts [52]. It has been seen as an unfavorable approach, although it has a positive outcome among EFL nursing students [53].

Seldom do researchers follow the studied groups to assess the long-term effects of the reading interventions [23]. The follow up time used in this study was one week. Daniel et al. [23] reported that among studies which embraced a follow-up time in data collection, the median of the follow-up time was 6 weeks. It is perhaps, recommended to have additional studies with greater time between short-term comprehension test and follow-up comprehension test to build on the current study’s findings.

According to the results, among the different demographic and background parameters, only gender differences were identified to have significant impact on reading comprehension with a favor to male in the long-term of reading comprehension impact of synthesization and female in the short-term reading comprehension impact of translation. Over decades, studies in reading comprehension have increasingly highlighted the importance of individual characteristics as significant contributing factors in the reading comprehension of college students [54]. Within these studies gender differences in reading comprehension was noticed. This is because gender acts as a powerful context in which the individual’s learning and development occurs [55]. Within such context, readers may receive the same reading instructions, however, gender imposes differences in attention, interest and activities preference [55].

The current study also investigates whether EFL readers’—nursing students—capabilities have a relationship with demographic data in the two groups regardless of the comprehension result. The results of the current study showed that the English proficiency effect was significant with determination, metacognitive and social skills in synthesization group (p = 0.00, p = 0.01, p = 0.007 respectively), indicating the interactive role of key reader capabilities and text-activities in reading comprehension. Researchers claimed that reading comprehension involves the interaction between the cognitive processes and skills of the reader and the linguistic characteristics of a text [54]. Indeed, reading comprehension is a complex cognitive process that combines several factors including several cognitive abilities [56]. In contrast, Al-Bidawi [57] found that Saudi undergraduate EFL students favored social skills over cognitive and meta-cognitive skills. According to linguistic scientists, during synthesization, students build a kind of cognitive schema with emphasis on reaching understanding [58]. Relevant prior knowledge includes not only knowledge about the text subject, but also knowledge of the purpose of reading and the use of reading strategies [59]. Taking in to account the text and readers factors, the finding may provide empirical evidence for using SBR when enhancing the scientific background to non-English speakers. The findings may support both the role of text-activities as well as the contributing factors for EFL nursing students’ reading comprehension of scientific research.

Finally, nursing academic educators should strive to deliver effective teaching strategies in order to enhance students’ scientific research comprehension process [60]. The literature has shown that nursing education for scientific research highlights text difficulties as the most important factor for the use of scientific research [27], however, implementing rigorous investigation processes to identify which reading interventions are most successful in making nursing students understand and enact scientific research is highly recommended [60].

## Implications

The current study findings have several implications for EFL—nursing students—nursing educators, program coordinators and relevant decision-makers. First, EFL—nursing students—must become aware of the impact of factors such as their cognitive, social, academic abilities, and English proficiency on their reading comprehension. Specific measures could be utilized so as to overcome the issues concerning these factors to enhance good outcomes in reading comprehension. Secondly, nursing educators should enhance students’ cognition and metacognition abilities to guess complicated medical terms from the contexts. They must also explore techniques to take advantage of the times when nursing students in clinical practice encounter clinical cases in which it would be helpful to use scientific research. The current study results suggest that nursing educators need to apply synthesizing reading strategies frequently in reading classes. In doing this, the nursing students will be equipped with such skills to overcome any issues in reading scientific research. Thirdly, program coordinators and relevant decision-makers must highlight the importance of providing nursing educators with competencies in terms of reading strategies by organizing training sessions and workshops. Consequently, nursing educators become more competent and more qualified in teaching the understanding of and the use of scientific research.

## Limitations

The current study has several limitations. First, a limited time period—only one session—was used in the current study. Indeed, one session of text-activities, around 2 hours, is not really a sufficient time to determine the predicted effect of both text-activities on EFL—nursing students. Perhaps more sessions and a longer period of time (whole semester) may have different results between text-activities and the association factors. Secondly, the study was limited to a particular geographic context in the Saudi Arabia. Thus, the results cannot be generalized to all EFL—nursing students. The impact of the social background of readers on the comprehension’s three main components need recognition. Thirdly, this is a two-group posttest-only randomized experiment design with no control groups or pretest. This interferes with hypothesis testing (if any). Fourth, the sample size was limited to 60 nursing students per group. Although this sample size is considered adequate based on the literature, the sample size also limited the generalizability of the findings. Future studies should employ a control group in addition to the text-activities groups as well as larger sample size and longer duration. Despite these limitations, the current study has effectively addressed several design concerns found in previous reading comprehension studies. Specifically, the current study used random sampling of nursing students to both groups, and treated both groups in the same way in terms of providing them the same scientific research material to read, and the same amount of time to finish the task. Both groups were kept unaware of the aim of the study so that their awareness would not affect the results of the experiment adversely.

## Conclusion

Conducted with an aim to assess which reading intervention produces long-term sustainability effects in scientific research reading comprehension, the present study found that the synthesization group significantly outperformed the translation group on both the short-term and long-term reading comprehension tests. An important strength of this study is that it investigated an area that has been overlooked for an extended period of time. Enhancement of EFL nursing students’ reading comprehension of scientific research requires selecting the appropriate reading intervention; better input should yield improved outcome. Synthesization can be a valuable tool in scientific research education if it is utilized in cooperation with rich clinical experience. In this sense, it is important that further studies be conducted in different contexts and at various levels to reveal the effect of synthesization on scientific research reading comprehension. More studies that cover a wider sample are required to understand how synthesization contributes to the comprehension process of scientific research in differing personal contexts.

## Supporting information

S1 Appendix Assessing the short-term reading comprehension outcome. (DOCX)

S2 Appendix Assessing the long-term reading comprehension outcome. (DOCX)

S1 Data (DOCX)
