# Supplementary material for: The sustainability effects of two reading interventions on Saudi nursing students’ comprehension of scientific research
Source: PLoS One. 2024 Oct 24;19(10):e0309898. doi: 10.1371/journal.pone.0309898 (PMC11500948; doi:10.1371/journal.pone.0309898)
Supplement: S1 Appendix — (DOCX) [file pone.0309898.s001.docx]

**S 1** Assessing the short-term reading comprehension outcome (S 1 Appendix)

Example 1: T/F Qs

State whether the following statement is true or false:

Some studies included in the review reported a significant reduction in the number of pressure ulcers in patients who were turned more frequently (every 2 to 3 hours) when using a standardized institutional mattress.

- 1. True
  2. False

Example 2: MCQ

From the following, select the correct statement

1. "The outcomes of repositioning patients on an alternating pressure air mattress (APAM) are better than those of repositioning patients on a high-density foam mattress or a pressure-reducing mattress."
2. "The outcomes of repositioning patients on a pressure-reducing mattress are better than those of repositioning patients on a high-density foam mattress."
3. "No difference was found between the outcomes of repositioning patients on an alternating pressure air mattress (APAM) and those repositioned on a high-density foam mattress or a pressure-reducing mattress."
