# Supplementary material for: The sustainability effects of two reading interventions on Saudi nursing students’ comprehension of scientific research
Source: PLoS One. 2024 Oct 24;19(10):e0309898. doi: 10.1371/journal.pone.0309898 (PMC11500948; doi:10.1371/journal.pone.0309898)
Supplement: S2 Appendix — (DOCX) [file pone.0309898.s002.docx]

**S 2** Assessing the long-term reading comprehension outcome (S 2 Appendix)

Case vignette

You have been assigned to a patient who has been bedbound for the last two weeks due to stroke complications. The patient has been receiving care using a standard hospital mattress with frequent turning (every 4 to 6 hours). During morning care, you observed skin discoloration on the patient’s bony prominences which indicates an early sign of bed ulcer development. You discussed your observation with your colleagues.

Questions:

From your recent reading, what is the most appropriate evidence-based measure you should recommend to reduce the risk of developing hospital-acquired pressure ulcers?

1. Consider doing more frequent turning 2- to 3-hourly instead of 4- to 6-hourly
2. Consider changing the standard hospital mattresses
