# Supplementary material for: The sustainability effects of two reading interventions on Saudi nursing students’ comprehension of scientific research
Source: PLoS One. 2024 Oct 24;19(10):e0309898. doi: 10.1371/journal.pone.0309898 (PMC11500948; doi:10.1371/journal.pone.0309898)
Supplement: S1 Data — (DOCX) [file pone.0309898.s003.docx]

| **Vocabulary Learning Strategies Questionnaire VLSQ** | | | | | | |
| --- | --- | --- | --- | --- | --- | --- |
| **Determination strategies: DET** | | **Never 0** | **Seldom 1** | **Sometimes 2** | **Often 3** | **Always 4** |
| DET1 | Use an English–Arabic dictionary to discover the meaning of new words. |  |  |  |  |  |
| DET2 | Use an Arabic–English dictionary to discover the meaning of new words. |  |  |  |  |  |
| DET3 | Use an English–English dictionary to find the meaning of new words. |  |  |  |  |  |
| DET4 | Guess the meaning from context to discover the meaning of new words. |  |  |  |  |  |
| DET5 | Guess the meaning from word classes, such as noun, verb, adjective, adverb, to discover the meaning of new words |  |  |  |  |  |
| DET6 | Guess the meaning by analyzing the structure of words (prefixes, roots, and suffixes) to discover the meaning of new words. |  |  |  |  |  |
| DET7 | Guess the meaning from grammatical structure of a sentence to discover the meaning of new words. |  |  |  |  |  |
| DET8 | Guess the meaning from aural features, such as stress, intonation, pronunciation, to discover the meaning of new words. |  |  |  |  |  |
| **Memory strategies: MEM** | | **Never** | **Seldom** | **Sometimes** | **Often** | **Always** |
| MEM1 | Categorize new words according to their synonyms and antonyms. |  |  |  |  |  |
| MEM2 | Group new words in relation to similar pronunciation and spelling. |  |  |  |  |  |
| MEM3 | Group new words together to learn new vocabulary. |  |  |  |  |  |
|  | Connect pictures to the meanings of new words. |  |  |  |  |  |
| MEM5 | Observe the parts of speech of the new vocabulary items. |  |  |  |  |  |
| MEM6 | Examine the new words’ affixes (prefixes and suffixes). |  |  |  |  |  |
| MEM7 | Use new vocabulary items in sentences repeatedly. |  |  |  |  |  |
| MEM8 | Use semantic maps to learn new words. |  |  |  |  |  |
| **Cognitive strategies: COG** | | **Never** | **Seldom** | **Sometimes** | **Often** | **Always** |
| COG1 | Use a new lexical item by writing it repeatedly in sentences. |  |  |  |  |  |
| COG2 | Repeat orally a single word with its meanings to learn it. |  |  |  |  |  |
| COG3 | Revise previous English lessons and take notes in class to learn the new vocabulary items. |  |  |  |  |  |
| COG4 | Practice orally new words with their lexical sets. |  |  |  |  |  |
| COG5 | Keep a notebook for a vocabulary list with meanings and examples to learn the new vocabulary items. |  |  |  |  |  |
| COG6 | Associate new vocabulary items with physical objects to learn the lexical items. |  |  |  |  |  |
| COG7 | Listen to vocabulary CDs to learn new vocabulary items. |  |  |  |  |  |
| COG8 | Write new lexical items with meanings on flash cards to learn them. |  |  |  |  |  |
| **Metacognitive strategies: MET** | | **Never** | **Seldom** | **Sometimes** | **Often** | **Always** |
| MET1 | Expand the knowledge of lexical items by listening to English songs. |  |  |  |  |  |
| MET2 | Learn new words by watching English-speaking movies with subtitles. |  |  |  |  |  |
| MET3 | Study new vocabulary items from advertisements, written signs, written notices, etc. |  |  |  |  |  |
| MET4 | Learn new lexical items by reading articles from several sources as magazines, newspapers, brochures, etc. |  |  |  |  |  |
| MET5 | Expand the knowledge of vocabulary items by testing your vocabulary knowledge with word lists. |  |  |  |  |  |
| MET6 | Learn new words by listening to English radio programs |  |  |  |  |  |
| MET7 | Expand the knowledge of lexical items by doing extra curriculum exercises from different sources, such as articles, texts, internet, etc. |  |  |  |  |  |
| MET8 | Learn new words by relating newly-learned words with previously learned ones. |  |  |  |  |  |
| **Social strategies: SOC** | | **Never** | **Seldom** | **Sometimes** | **Often** | **Always** |
| SOC1 | Ask instructors of English for Arabic translation of new lexical items. |  |  |  |  |  |
| SOC2 | Communicate with instructors of English in English to use a new lexical item in a sentence to increase the knowledge of vocabulary. |  |  |  |  |  |
| SOC3 | Communicate with instructors of English in English to ask for a synonym of a new word or to explain it. |  |  |  |  |  |
| SOC4 | Look for extra English information through the Internet to learn new vocabulary items. |  |  |  |  |  |
| SOC5 | Discuss in English with classmates to know and expand the meaning of a new vocabulary item. |  |  |  |  |  |
| SOC6 | Communicate with foreigners in English through different types of media to develop new vocabulary. |  |  |  |  |  |
| SOC7 | Play English games, such as scrabble, crossword puzzles to find meaning of a new vocabulary item through group work activity. |  |  |  |  |  |
| SOC8 | Study and practice meaning of new vocabulary items in-group to expand lexical knowledge. |  |  |  |  |  |
